# A free lunch or a walk back home? The school food environment and dietary behaviours among children and adolescents in Ghana

**DOI:** 10.1007/s12571-017-0712-0

**Published:** 2017-10-01

**Authors:** Meenakshi Fernandes, Gloria Folson, Elisabetta Aurino, Aulo Gelli

**Affiliations:** 1 Partnership for Child Development Imperial College London, Department of Infectious Diseases, London UK; 2 University of Ghana Noguchi Memorial Institute for Medical Research (NMIMR), Department of Nutrition, Accra, Ghana; 3 International Food Policy and Research Institute (IFPRI), 2033 K St, NW, Washington, DC 20006-1002, USA

**Keywords:** Food environments, Food Access, Dietary quality, School meals, Children and adolescents

## Abstract

Food environments can play an important roles in shaping nutrition and health outcomes. One such environment that has potential to affect youth is the school food environment. In contrast to higher-income countries, however, there is a critical evidence gap on the role of school food environments on children and adolescents in low- and middle-income countries. This mixed-methods study contributes to filling this gap by investigating the role of school food environments on dietary behaviours of children and adolescents in Ghana. It draws on data from household and school questionnaires as well as focus group discussions collected as part of the baseline for an impact evaluation of the Ghana School Feeding Programme (GSFP).

Multi-level regression models were fitted with random intercepts at the individual, household and community levels. Excerpts from the focus group discussions provided a deeper understanding of quantitative findings. Children and adolescents who received free school meals provided by the GSFP or who lived further away from school were less likely to go home for lunch. More than half of sampled schools reported offering foods for sale by independent vendors, the most common being meals followed by confectionery, fruit and sugar-sweetened beverages. Predictors of bringing money to school to buy food included non-receipt of free school meals, adolescence, greater commuting distance from home, household asset score, and urban location. Policy efforts focusing on the school food environment may contribute to healthy dietary behaviours for children and adolescents with positive impacts over the lifecourse.

## 1. INTRODUCTION

Food choices are mediated by a wide range of individual and environmental factors that include food availability and accessibility, social and peer influences, and tastes and preferences (Herforth and Ahmed 2015). The confluence of environmental factors in settings such as the home, community, school and workplace can define distinct food environments (Story et al. 2008). Interventions and policies that shape food environments hold promise for promoting healthy diets (CDC 1996; Hawkes et al. 2015). The school food environment has been recognized as an entry point to support healthy food choices among children (De Villiers & Fabier 2015).

The present study investigates the role of the school food environment on dietary behaviours among children and adolescents in the context of Ghana, a country undergoing the nutrition transition. In doing so it seeks to follow the definition of food environment presented by Herforth and Ahmed (2015). Quantitative data from national survey data primarily shed light on how availability and affordability might shape behaviours. The qualitative data from focus group discussions provide insights into these dimensions as well as convenience and desirability.

The national school feeding programme, which provides free meals to selected schools, is a defining feature of the school food environment in Ghana and many other countries in the world (Bundy et al. 2009). The findings related to school meals may be especially relevant for the prevention of malnutrition and diet-related diseases over the lifecourse (Bundy et al. 2009).

### 1.1 School food environments in high-, middle- and low-income countries

Research from high-income countries indicates that schools are an important food environment for children and adolescents (Kubik et al. 2003; Wechsler et al. 2000). In particular, school feeding programmes, which provide food to children in schools on a regular basis, can contribute to improved diets and health (USDA, 2012; Story et al. 2002). Food advertising and placement, nutrition and health education, sales of meals and snacks by independent vendors and peer influences may also play a role (Story et al. 2002).

Less is known about school food environments in low- and middle-income countries, especially in sub-Saharan Africa. The bulk of evidence has been generated in South Africa (Claasen et al. 2016; Faber et al 2014; Jacobs et al. 2013; Meko et al 2015; Oosthuizen et al, 2011). For example, one study highlighted a number of issues in the school food environment such as the low content of fruits and vegetables in school meals, that about a quarter of the students did not eat breakfast, and the wide consumption of unhealthy food items bought in nearby tuck shops and by vendors located in the schools (Faber et al 2014). One study presents a qualitative conceptual framework for healthy eating among adolescents in Ecuador (Vepsalainen et al 2015). Studies investigating the impact of school-based interventions on dietary behaviours have been undertaken in Brazil (Gaglianone et al. 2006; Sichieri et al. 2009; Vargas et al. 2011), Thailand (Banchonhattakit et al. 2009), Trinidad and Tobago (Francis et al. 2010). The measures of dietary behaviours employed in these studies primarily included knowledge and attitudes about healthy eating, as well as consumption of energy-dense foods such as soft drinks and fast-food.

The bulk of the literature from low- and middle-income countries regarding the school food environment focuses on how the provision of school feeding may impact intra-household food reallocation (Greenhalgh et al. 2007). The provisions of food may represent a significant transfer to households and the evidence suggests that households do not respond by providing the child with less food at home, a finding known as the ‘flypaper effect’ (Greenhalgh et al. 2007). Fewer studies have investigated other dietary behaviours such as bringing money to school to buy food and the types of foods purchased. This behaviour may be associated with the consumption of less healthy foods and worsen overall dietary quality, especially with the widespread shift from under- to over-nutrition and related non-communicable diseases known as the nutrition transition (Popkin 1998). In one study from Jamaica, children who received school meals were as likely to bring money to school as children who did not receive school meals, however purchase patterns were not analysed (Powell et al. 1998). More broadly, several reviews of the literature find that school feeding can contribute to nutrition, health and educational outcomes for children and adolescents (Snilstveit et al 2015; Kristjansson et al, 2015).

More research in low- and middle-income countries is merited as the school food environment may offer promising policy levers to counteract the nutrition transition (Popkin 1998). Studies have documented increasing rates of overweight and obesity among children and adolescents in many countries alongside a persistently high prevalence of undernutrition and micronutrient deficiencies (Ng et al. 2014; De Onis et al. 2010). The overall shift from under-to over-nutrition may be driven in part by altered dietary behaviours, which may be developed during adolescence and endure over the lifecourse (Popkin 1998; Dunn et al. 2000; Mikkilä et al. 2005; Aurino et al. 2016).

### 1.2 The school food environment analytical framework

Our multi-methods study was guided by the framework set by Herforth and Ahmed (2015), which defines food environments as the “availability, affordability, convenience, and desirability of various foods.” For children and adolescents who attend schools, the school food environment may wield significant influence as has been demonstrated in high-income countries (Wechsler, 2000). Previous studies have established approaches to define, measure and analyse these dimensions using ecological models and social cognitive theory (Glanz 2005; Lytle 2009; Story et al. 2008).

Food supplied by school feeding programmes and independent vendors reflect the availability dimension, which may depend on community characteristics such as urbanicity. Affordability is reflected in the cost of these foods in relation to the household income of children and adolescents. Convenience is also likely to influence decision-making, especially for children who live further away from school. Lastly, desirability may be enhanced by factors shaping preferences such as cultural norms that may be established by food consumption patterns in the community and by peers, as well as nutrition education and advertising. Herforth and Ahmed (2015) note that existing quantitative measures relate to the availability and affordability dimensions of the food environment. There is a need to consider approaches for measurement that reflect all four dimensions. Previous studies have highlighted that taste, habit strength and self-efficacy are important factors for children and adolescents in some contexts (Verstraeten et al. 2014).

## 2. METHODS

This mixed methods study draws on quantitative and qualitative data from surveys and focus groups. The data collection took place during 2013 and 2014 as part of the baseline for an impact evaluation of the Ghana HGSF programme that sought to assess the effect of the programme on a wide range of child and community outcomes, including education, nutrition and agriculture (Gelli et al, 2016).

### 2.1 Study context

Ghana was a lower-middle income country at the time the study was conducted. The population includes more than nine major tribes or ethnic groups with over 100 sub-groups. Members of the various ethnic groups share a common cultural heritage, history, language, and origin. The 10 regions of Ghana correlate to some degree with ethnic groups, and can generally be grouped into the North (Upper East, Upper West and Northern regions), which is generally less economically developed than the South (anywhere from the Brong Ahafo Region southwards). Ghana launched a school feeding programme in 2005 that sought to provide a daily, hot meal to children attending selected schools. In 2012, it was estimated that one in three children attending public, primary schools benefited from the programme. The model is known as home-grown school feeding (HGSF) as foods are procured from the community with the objective of promoting the incomes of local smallholder farmers, as well as the nutrition of children. The meals, which typically include a locally produced staple such as rice, cassava, or yam with a local side dish such as *groundnut* soup or *okro* stew^1^, are intended to be well-balanced and appeal to local tastes and preferences (Parish and Gelli, 2015). In 2012, the Ghana School Feeding Programme (GSFP) was retargeted to districts with the highest levels of food insecurity and poverty (Gelli A. et al. 2016).

### 2.2 Study population

Nutrition indicators among children and adolescents are poor in Ghana, reflecting the triple burden of malnutrition. In addition, there is significant regional variation, particularly between the South and North (Agbozo et al 2016; Prince and Laar 2014; Owusu et al. 2014). About one out of five school-age children in Ghana were estimated to be moderately or severely stunted (Manyanga et al. 2014). On the other hand, overweight is on the rise, especially among urban children attending private schools, although the exact figures are not clear (Agbozo et al 2016). Some studies suggest that the prevalence of obesity in the population is about one to four percent (Abiba et al. 2012; Muthuri et al. 2014). Another study from secondary school students found a prevalence of overweight and obesity of 13.3 percent among girls and 6.7 percent among boys (Manyanga et al. 2014). Micronutrient deficiencies are widespread as well. Among rural schoolchildren in Northern Ghana the prevalence of anaemia was estimated to be 64 percent (Abizari et al 2012). Another study reported that 44 percent of adolescent girls suffered from anaemia (UNICEF 2012). Another investigation using the data from the present study found that average BMI-for-age z-score was -0.592 while the average height-for-age z-score was -0.925 (Gelli A. et al. 2015).

### 2.3 Study sample

The sample for the study was based on an impact evaluation (Gelli A. et al, 2016). From a set of 60 of the total of 216 districts in Ghana, two comparable public primary schools and the surrounding communities in each district were selected.^2^ Household listings were compiled in each enumeration area (EA) by the survey team supervisors assisted by community leaders. Maps were obtained for most of the EAs from the Ghana Statistical Service. The EA maps made it possible to identify all dwelling structures within a geographical space with a well-defined boundary. All dwelling/housing structures within each EA were serially numbered to facilitate the complete listing of households. The list of all households with a child aged five to 17 years of age in each EA constituted the sampling frame from which participating households were selected at random for the household questionnaire. About 20 to 25 households were selected from each community for the survey. The school questionnaire was administered in each of the selected schools. Household and school questionnaires were administered by teams of enumerators from the University of Ghana in the local language. Questionnaire responses were input and cleaned at the University of Ghana.

In addition to the household and school survey, focus group discussions were undertaken in nine communities (nine focus groups in total) between March and April 2014. The focus groups were moderated by four trained and experienced data collectors who had no established relationship with participants prior to the study. The discussions were conducted in the predominant language of the community. Interviews, each of which lasted about an hour and a half on average, were audio recorded and transcribed verbatim into English upon return from the field.

While the intention for the impact evaluation was to field the questionnaire prior to the introduction of the GSFP, the data indicated that several schools had already started to provide school meals. Although this constituted a challenge for the impact evaluation, it is a feature of the data that was exploited for the present study.

### 2.4 Household and school questionnaires

The household and school questionnaires were based on tools previously used in country or in a similar impact evaluation developed in Mali (Masset and Gelli, 2013). The school questionnaire was adapted to Ghana with the support of the GSFP monitoring and evaluation team. Household agriculture, food consumption and expenditure modules were based on the Living Standard Measurement Study (LSMS) undertaken in Ghana, as were the education participation modules (LSMS, 2009-2010). TABLE 1 presents the variables from the household and school questionnaires that were used for the quantitative analysis. ANNEX TABLE 1 presents the wording from the questionnaires of the school food environment questions.

**TABLE 1:** Variables used in the quantitative analysis

Household questionnaire	
Child/adolescent dietary behaviours:	Had breakfast at home
	Went home for lunch
	Took food home from school
	Received free meal at school
	Brought food from home
	Brought money from home
	Spent money at or near school
Child/adolescent characteristics:	Age
	Gender
	Ethnicity
	Traveling time to school
Household characteristics:	Household size
	At least one sibling less than 5 years of age
	Asset score
	Education level, head of household
	Gender, head of household
Community characteristics:	Rural/urban
	Region

School questionnaire	
Food environment:	Participation in the GSFP
	Types of foods sold in school

The respondent to the household questionnaire was the head of household or caregiver. As the measures of dietary behaviours were not reported by the children and adolescents themselves, the responses were considered as perceptions. In the school questionnaire, head teachers or caterers reported whether or not the school was in the GSFP, and the availability of certain foods for sale at the school by independent food vendors. The types of food reported for sale were meals, confectionery, fruit and sugar-sweetened beverages.

The household questionnaire included a number of modules. In the education module, the respondent reported the dietary behaviours of the child or adolescent during the past week. If the child or adolescent attended school, the respondent was asked if he/she received free meals at school. If the response was positive, she/he was asked how many days a meal was received by the child or adolescent in the past week and whether they consumed less food at home on the days on which they received a free meal at school. The household head was also asked to report the number of days in the past week that the four dietary behaviors were exhibited, as well as the amount of money spent on food at or near school. The number of days the dietary behavior occurred during the past week ranged between zero and five days. With regards to money taken to school, a few extreme values greater than 20 Ghana cedis (GHS) (equivalent to about $10 at the time of the survey) were noted and recoded as missing. Caregivers also reported if the child or adolescent received free meals at school.

The set of variables also included other individual, household and community characteristics that may be important determinants of dietary behaviours such as age and gender (Glanz 2005; Aurino et al 2016). For some studies, childhood and adolescence were defined in terms of biological growth and puberty onset, while for others the definition was in terms of psychosocial growth transitioning to adulthood (Dehne and Riedner 2001; Kroger 2003). The latter definition was used for the study and corresponds to the age-appropriate thresholds for basic education or primary school, junior high school and senior high school in Ghana (Akyeampong et al. 2007). Those aged five to 10 years of age were classified as children, while those between 11 and 14 were classified as young adolescents, and 15 to 17 years olds were classified as older adolescents.

Several variables reflect household socio-economic status (SES), which may also be an important determinant of dietary behaviours (Aurino et al 2016; Vepsalainen et al 2015). These variables include the gender and education level of the household head, as well as household asset score. An asset-based index, based on ownership of durables and access to services, was constructed using principal components analysis and quintiles (Filmer and Pritchett, 2001). This measure relates to the affordability dimension of the school food environment, as do household size and the presence of a sibling less than five years of age, which may reflect increased demands on household resources.

The time and distance to travel to and from school was also reported in the household questionnaire. Children and adolescents who live further away from school may benefit more from having free school meals or foods offered for sale than children who live closer and who can go home more easily for lunch, reflecting the convenience dimension of food environments. Commuting time was chosen for the analysis as it was expected to play a stronger role in shaping these dietary behaviours. Distance in kilometers, which was also reported in the survey, was tested in the sensitivity analysis. Values that exceeded four hours were recoded as missing. Households were classified as living in urban areas according to enumeration maps (Ghana Statistical Service 2012). Urban areas included the national capital Accra as well as some district capitals, and may relate to all dimensions of the food environment. Following the Herforth and Ahmed (2015) framework, TABLE 2 presents an overview of the dimensions of the school food environment and related individual and household characteristics. Variables represented in the present analysis are noted.

**TABLE 2:** Themes and sub-themes of focus group discussions

Main theme	Sub-themes
Strategies families use to keep children healthy	Education, healthcare, hygiene and nutrition.
Feeding strategies used to keep children healthy	Various food groupings and their health benefits for children; Food hygiene; Provision of meals at home to ensure children eat well, as opposed to buying street food, parental responsibility.
Household feeding	Factors influencing food allocation such as age, gender, monetary contribution, and health status; Advantages and disadvantages of the school feeding programme from the perspective of the children and caregivers; How school feeding may have influenced mode of feeding at home or not.
Vitamins and food fortification	Awareness of food sources of vitamins; Access and barriers to these foods, and their roles in the diet; Familiarity with commercially packaged vitamin supplements, the concept of food fortification and openness to the concept of the use of MNPs in school meals.

Note: Main themes were defined before the data collection while sub-themes emerged after review of the transcripts.

### 2.5 Survey analysis

The quantitative analysis used multi-level models with random effects at the household, school and district levels to explore how factors at different levels of influence including the school food environment shaped individual child and adolescent dietary behaviours (Sniders and Bosker, 1999).^3^ Drawing from the available measures in the household questionnaire, the dependent variables in the analysis were: (1) having breakfast at home; (2) going home for lunch; (3) bringing food to school; and (4) spending money on food at or near school. The receipt of free school meals reported in the household questionnaire was a key independent variable. The presence of GSFP in the school as reported in the school questionnaire was excluded from the list of independent variables in the estimations due to multicollinearity, while the availability of foods for sale by independent vendors as reported in the school survey was included in the list of independent variables. In addition, the model controlled for moderating individual and household factors at the different levels through the addition of fixed effects. The inclusion of fixed effects in the models was tested with the log-likelihood ratio test. Several sensitivity analyses were undertaken for the multivariate findings. Binary constructions of the outcome variables were also tested which noted if the diet behavior occurred at least once in the past week. In addition, alternative age groupings for childhood and adolescence were tested to investigate the robustness of findings.

The analytic sample was defined as follows. First, the sample was limited to youth aged five to 17 years who reported still being in school and attended kindergarten up to sixth grade in a primary or public school at least one day in the previous week. The sample was then restricted to children and adolescents who reported whether or not they received a free school meal at least once in the previous week as well as other dietary behaviours. Respondents with missing information were more likely to be from urban areas, but otherwise did not significantly differ from non-respondents. Observations with missing values for other variables from the household and school survey were also excluded.

In total, the analytic sample included 4,258 children ages five to 17 years from 1,951 households located in 111 communities that corresponded with 111 schools sampled in the school survey. Almost all children and adolescents in the analytic sample (99 percent) attended full-day school without separate morning and afternoon shifts, and of these, 97 percent attended school four or five days of the previous week.

## Focus group methods

The focus groups were conducted using standard procedures (Tong et al. 2015). The interview guide for the discussions was adapted from a Focused Ethnographic Study tool developed by the Nutrition Department, NMIMR, Ghana and Cornell University and previously used in Ghana (Pelto et al. 2013). The guide is framed within the social-ecological model for the determinants of nutrition. Some of the modules were retained while some areas of investigation were added.

All caregivers of child and adolescent study participants (who had been previously selected from the communities for the baseline survey for the impact evaluation) were invited to participate in the focus group discussions. The discussions took place in a quiet area outside on the school premises where distractions were minimal. On average, there were eight to ten participants per focus group discussion and included both males and females. No limitations were noted for the joint participation of men and women in the same focus group discussion. The experiences and views of caregivers were solicited regarding the implementation of a complex intervention with a focus on health and diets. Discussions around specific questions continued until no new information arose. Each participant was given two cakes of soap in appreciation for their time.

### 2.6 Transcript analysis

Four main themes were constructed by a qualitative researcher using basic content analyses through a deductive approach. Responses to specific questions were grouped and analyzed for emerging themes and sub-themes. Similarities and differences across the groups were noted. The researcher coded the transcripts using NVivo software, ensuring consistency across the various transcripts. TABLE 2 presents the themes and sub-themes of the focus group discussion. The data was written up under the defined themes and sub-themes making full and appropriate use of quotations that illustrated the nature of the interactions observed. Two themes – (1) Feeding strategies to keep children healthy; and (2) Household feeding – were the focus of the present study.

Findings for these two themes were reviewed against findings from the quantitative analysis, similar to Morrow et al. (2014) and Aurino and Morrow (2015). To the degree possible, some excerpts from the focus group discussions were used to shed light on possible reasons behind findings from the quantitative analysis. Participant quotations were used to note findings from the focus group discussions. The community where the participant quotations were gathered were noted, while no individual identifying information is given.

## 3. RESULTS

### 3.1 Descriptive statistics from the surveys

TABLE 3 presents descriptive statistics of the children and adolescents sampled from the household survey. These variables served as covariates in the subsequent multivariate analysis. About half of the sample was female while about 40 percent were adolescents (aged 11 to 17 years). The distribution of the sample by region reflects the sampling strategy of the impact evaluation, which concentrated in the north where food insecurity and poverty rates were higher. The three main ethnic groups were Gurma (36 percent), Akan (21 percent) and Mole-Dagbani (24 percent). Average household size was seven people while about 60 percent had at least one sibling less than five years of age. About three percent of children lived in urban areas while the average commuting time to and from school was 16 minutes. About 20 percent of children and adolescents received a free school meal in the past week, 94 percent of whom received free meals four or all five of the school days in the past week.

**TABLE 3:** Descriptive statistics of analytic sample from the household survey (N=4,258763)

Categorical variables:		%	Obs (n)
Age group^a^	5–10 years old	61.2	2,607
	11–14 years old	32.4	1,358
	15–17 years old	6.4	273
Female		46.9	1,997
Ethnic group	Akan	20.7	881
	Ga-Dange	1.9	79
	Ewe	7.6	325
	Guan	1.4	61
	Gurma	35.6	1,515
	Mole Dagbani	24.1	1,027
	Grusi	3.5	148
	Mande	1.6	67
	Other	3.6	155
Region	Western	3.4	160
	Central	3.3	158
	Greater Accra	0.7	31
	Volta	8.9	414
	Eastern	4.8	221
	Ashanti	11.2	523
	Brong Ahafo	11.8	566
	Northern	27.7	1,291
	Upper East	15.6	727
	Upper West	12.1	564
Urban		2.8	118
Education level of household head^b^	Some or no formal schooling	64.7	2,755
	Some secondary school	32.4	1,381
	Vocational education or college	2.9	122
Female head of household		16.8	716
At least one sibling less than 5 years of age^c^		62.1	2,646
Received free school meal			
	At least once in past week	22.3	950
	Number of days in the past week	4.6	950

Continuous variables:	Mean	Range
Commuting time to and from school (min)^d^	16.0	1–240
Household asset score^e^	-0.1	-1.1–5.1
Household size	6.9	2–20

a Reference group is children ages 5 to 10 years of age. Young adolescents are 11 to 14 years old and older adolescents are 15 to 17 years old.

b A sibling 5 years of age or less.

c Commuting distance to and from school in minutes.

d Household respondent reported level of education achieved of each household member including household head. Reference group is none or some formal schooling.

e Estimated household assets. Calculated using principal components analysis based on reported ownership of durables and access to services.

TABLE 4 presents descriptive statistics of reported dietary behaviours during the past week from the household survey. Most children and adolescents (91 percent) had breakfast at home most of the days of the past week, while a lower but still substantial share of children and adolescents had lunch at home (62 percent). Some reported bringing food to school (about five percent) although it was more common to spend money on food at or near school (40 percent). These children and adolescents brought an average of GHS 4.40 ($2.20) to school the past week, with 45 percent bringing more than the weekly equivalent of the GSFP subsidy of GHS 2.50 ($1.75).

**TABLE 4:** Dietary behaviour patterns reported by children and adolescents (N=4,258)

	At least once in past week ^a^		Average number of days in past week ^b^	
	%	Obs (n)	Mean	Obs (n)
Had breakfast at home	90.6	3,857	4.5	3,857
Went home for lunch	62.4	2,655	4.5	2,655
Took food to school	4.8	203	3.2	203
Took money to school	39.5	1,683	4.4	1,683

N/A = not applicable. Obs (N) = number of observations reporting in the affirmative.

a Reference period is past week.

b Average number of days ranges from 0 to 5 days;

c Question posed only to children and adolescents who reported receiving school meals at least once in the past week. The frequency of its occurrence was not reported.

**Figure 1:**
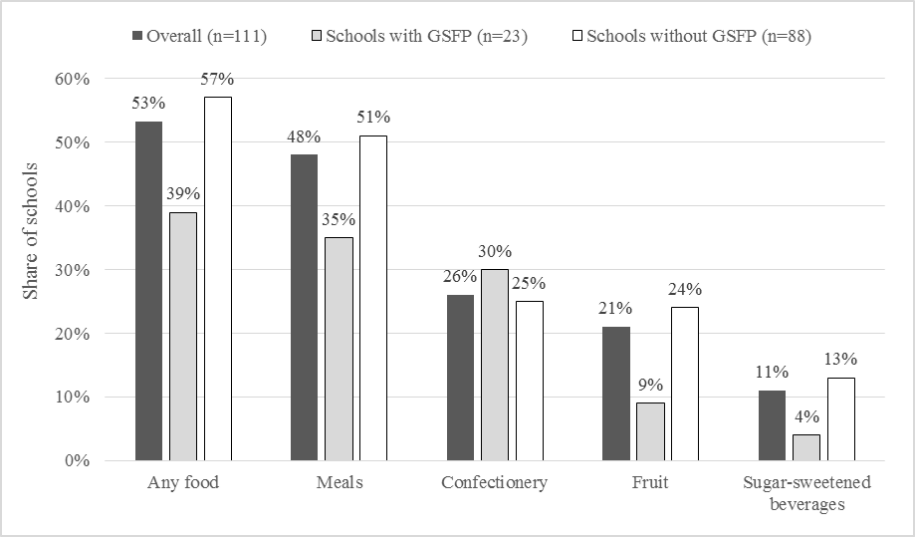
Availability of foods for sale in schools (N=111)

FIGURE 1 presents characteristics of the school food environment based on responses from the school questionnaire. Independent vendors offered foods for sale in more than half (53 percent) of schools. Availability of foods for sale through vendors appeared to be more common in schools without the GSFP (57 versus 39 percent), however, these differences were not statistically significant, perhaps due to a limited sample size.

The most common foods sold were meals (48 percent), followed by confectionery (26 percent), fruit (21 percent), and sugar-sweetened beverages (11 percent). An estimated 19 percent of schools reported vendors selling one option, the most common being meals, while 18 percent sold two items, the most common being meals and snacks or meals and fruit, and 14 percent sold three items, the most common being meals, confectionery and fruit. Just two schools reported vendors selling all four options – meals, confectionery, fruit and sugar-sweetened beverages – one of which was located in Greater Accra.

### 3.2 Multi-method analysis findings

This section presents the joint findings from the multi-level regression analysis as well as the analysis of the focus group transcripts. The findings are organized by dietary behaviour. Full results from the multi-level regression analysis can be found in TABLES 5 and 6. The estimated Intra-Class Correlations (ICCs) from these models indicated significant clustering at the district, school and household levels, with the greatest level of clustering at the household level ranging from 0.51 to 0.72. The fit of the models did not increase substantially with the inclusion of fixed effects, although it did improve relative to the empty models as indicated by log-likelihood ratio values.

**TABLE 5:** Multi-level regression results testing the associations between the school food environment and dietary behaviours (n=4,258)

	Days had breakfast at home^a^			Days went home for lunch^a^		
	Coeff	(SE)		Coeff	(SE)	
Days free school meal received at school^b^	-0.01	(0.01)		-0.04	(0.01)	***
Foods offered for sale in school^c^:						
Meals	0.07	(0.13)		-0.10	(0.20)	
Confectionery	0.18	(0.13)		-0.14	(0.21)	
Soft drinks	0.01	(0.17)		-0.53	(0.27)	*
Fruit	0.13	(0.17)		-0.32	(0.27)	
Female	0.01	(0.01)		0.03	(0.02)	
Age^d^:						
Young adolescent	-0.03	(0.01)	*	-0.08	(0.02)	***
Older adolescent	-0.08	(0.03)	**	-0.08	(0.04)	**
Ethnic group (ref=Akan)						
Ga-Dange	0.22	(0.27)		0.69	(0.39)	
Ewe	0.16	(0.16)		0.28	(0.24)	
Guan	0.11	(0.28)		0.05	(0.41)	
Gurma	0.22	(0.15)		-0.06	(0.22)	
Mole Dagbani	0.23	(0.15)		-0.21	(0.22)	
Grusi	0.50	(0.25)	*	0.04	(0.37)	
Mande	-0.30	(0.28)		0.13	(0.41)	
Other	0.19	(0.21)		0.11	(0.31)	
Household size	-0.02	(0.01)		-0.01	(0.02)	***
Young sibling^e^	0.06	(0.07)		-0.06	(0.10)	
Commuting time to school^f^	-0.00	(0.00)	***	-0.01	(0.00)	***
Household head level of education^g^:						
Some secondary school	-0.08	(0.08)		-0.02	(0.11)	
Vocational education or college	-0.03	(0.18)		-0.71	(0.26)	**
Female head of household	-0.10	(0.09)		-0.12	(0.13)	
Household asset score^h^	0.05	(0.03)		0.00	(0.04)	
North (ref = South)	0.56	(0.17)	*	0.69	(0.31)	*
Urban (ref = Rural)	0.04	(0.25)		-0.33	(0.40)	
Constant	3.61	(0.19)	***	2.98	(0.31)	***
Random effects						
District ICC:	0.11	(0.03)		0.20	(0.04)	
Community ICC:	0.01	(0.01)		0.02	(0.01)	
Household ICC:	0.83	(0.03)		0.74	(0.04)	
LL with fixed effects:	-4680			-6056		
LL from empty model:	-4723			-6171		

N=4,258 for all regressions. Statistically significant coefficients noted as follows: *p<0.05, **p<0.01, ***p<0.001. Coeff = coefficient; SE= standard error; LL= Log-likelihood ICC=Intra-class correlation.

a Dependent variables range from 0 to 5 days and reference period is previous week

b Reported in household survey. Ranges from 0 to 5 days and reference period is previous week.

c School food environment variables reported in the school survey.

d Reference group is children aged 5 to 10 years. Young adolescents are 11 to 14 years old and older adolescents are 15 to 17 years old.

e A sibling 5 years of age or less.

f Commuting distance to and from school in minutes.

g Household respondent reported level of education achieved of each household member including household head. Reference group is none or some formal schooling.

h Estimated household assets. Calculated using principal components analysis based on reported ownership of durables and access to services.

**TABLE 6:** Multi-level regression results testing the associations between the school food environment and dietary behaviours (cont’d)

	Days brought food to school^a^ (n=4,258)			Days brought money to school^a^ (n=4,258)			Amount of money (GHS) brought per week (n=1,604)		
	Coeff	(SE)		Coeff	(SE)		Coeff	(SE)	
Days free school meal received at school^b^	-0.00	(0.01)		-0.07	(0.02)	***	-0.08	(0.03)	**
Foods offered for sale in school^c^:									
Meals	0.04	(0.08)		0.79	(0.10)	***	-0.18	(0.29)	
Confectionery	-0.10	(0.08)		-0.24	(0.20)		-0.17	(0.27)	
Soft drinks	0.04	(0.11)		0.63	(0.26)	*	-0.11	(0.33)	
Fruit	0.01	(0.09)		0.40	(0.25)		0.07	(0.32)	
Female	0.00	(0.01)		-0.01	0.02)		-0.05	(0.04)	
Age^d^:									
Young adolescent	-0.01	(0.01)		0.04	(0.02)		0.22	(0.04)	***
Older adolescent	-0.03	(0.01)	*	0.15	(0.04)	***	0.65	(0.08)	***
Ethnic group (ref=Akan)									
Ga-Dange	-0.18	(0.15)		-0.75	(0.35)		0.70	(0.58)	
Ewe	-0.03	(0.10)		-0.28	(0.22)		-0.61	(0.33)	*
Guan	0.02	(0.16)		-0.29	(0.37)		-0.25	(0.60)	
Gurma	0.13	(0.08)		-0.47	(0.20)		-0.40	(0.31)	
Mole Dagbani	0.15	(0.08)		-0.05	(0.20)		-0.30	(0.30)	
Grusi	0.11	(0.14)		-0.92	(0.33)	**	-1.21	(0.62)	*
Mande	0.02	(0.16)		0.34	(0.36)		0.68	(0.51)	
Other	0.16	(0.12)		-0.19	(0.28)		0.36	(0.43)	
Household size	-0.00	(0.01)		-0.03	(0.02)		0.10	(0.04)	**
Young sibling^e^	0.05	(0.04)		-0.01	(0.09)		-0.09	(0.14)	
Commuting time to school^f^	0.00	(0.00)		0.01	(0.00)	***	0.00	(0.00)	***
Household head level of education^g^:									
Some secondary school	0.01	(0.05)		0.13	(0.10)		-0.11	(0.17)	
Vocational education or college	-0.13	(0.11)		0.40	(0.24)	*	0.53	(0.36)	
Female head of household	0.09	(0.05)		0.25	(0.12)		0.35	(0.18)	*
Household asset score^h^	0.02	(0.02)		0.16	(0.04)	***	0.17	(0.06)	**
North (ref = South)	-0.06	(0.08)		-0.84	(0.25)	***	-1.00	(0.34)	***
Urban (ref = Rural)	0.49	(0.15)	***	0.62	(0.36)	*	1.18	(0.46)	*
Constant	0.02	(0.10)		1.65	(0.27)	***	2.79	(0.41)	***
Random effects									
District ICC:	0.00	(0.00)		0.14	(0.03)		0.15	(0.05)	
School ICC:	0.09	(0.02)		0.03	(0.02)		0.04	(0.04)	
Household ICC:	0.86	(0.00)		0.77	(0.03)		0.74	(0.04)	
LL with fixed effects:	-2081			-6054			-2592		
LL from empty model:	-2096			-6196			-2732		

Statistically significant coefficients noted as follows: * p<0.05, ** p<0.01, ***, p<0.001. Coeff = coefficient; SE= standard error; LL= Log-likelihood; ICC=Intra-class correlation; GHS = Ghana cedis.

a Dependent variables range from 0 to 5 days and reference period is previous week

b Reported in household survey. Ranges from 0 to 5 days and reference period is previous week.

c School food environment variables reported in the school survey.

d Reference group is children ages 5 to 10 years of age. Young adolescents are 11 to 14 years old and older adolescents are 15 to 17 years old.

e A sibling 5 years of age or less.

f Commuting distance to and from school in minutes.

g Household respondent reported level of education achieved of each household member including household head. Reference group is none or some formal schooling.

h Estimated household assets. Calculated using principal components analysis based on reported ownership of durables and access to service.

#### 3.2.1 Dietary behaviours – breakfast at home

TABLE 5 indicates that the receipt of free school meals was not predictive of having breakfast before school, nor was the availability of foods offered for sale. In the focus group discussions, caregivers noted that children and adolescents typically had breakfast porridges prepared from cereals such as rice, maize and millet prepared by caregivers before leaving for school. Other foods mentioned include tea, rice, yam slices, beans and stew, which may be left over from the previous evening’s meal. For example,

‘Before they go to school, I give them porridge.’ (Caregiver from Ashanti Region); and ‘In the morning if there is no food in the house, I will buy porridge and bread. This can keep them till midday.’ (Caregiver from Central Region)

#### 3.2.2 Dietary behaviours – going home for lunch

The receipt of free school meals, however, was associated with having lunch at home less frequently (coeff=- 0.04, p<0.001). The focus group discussions suggest that children and adolescents in Ghana may have three or four meals a day, depending on whether the free school meals substituted for a meal prepared at home:

‘Some parents feed their wards three times a day…the children sometimes take lunch at home apart from the school meal (making four meals a day)’ (Caregiver from Volta Region).

Children and adolescents who received free school meals may come home less frequently at break time, but may still have food upon return from school at the end of the school day. While this was not ascertained in the questionnaire, it was raised in the focus group discussions. For example,

‘[The school meals programme] has a lot of benefits… the children used to come home to eat during break, and after eating, they will go and be hanging around the community, whilst we thought he or she has gone to school. You will come out later and find him or her still hanging around our homes instead of going back to school. Now that the children do not come home during break time to eat, we do not find them sitting or roaming at home when they are supposed to be in school (Caregiver from Northern Region).

The provision of free school meals may also reduce the practice of leaving school to search for food from other sources in the communities. For example,

‘Pupils no longer go to the market or bush looking for shea fruits and other wild fruits in the name of hunger. They know that they have food at school and if they are not there, they will not be served and so they are always ready with their bowls and spoons waiting to be served.’ (Caregiver from Northern Region).

#### 3.2.3 Dietary behaviours – eat less at home

Among those who received free school meals at school as reported in the household survey, an estimated 17 percent reported eating less at home. Discussions with the focus groups highlighted possible reasons for this finding, which included financial difficulties and that children were less hungry as they had eaten in school. This is shown in the quotes below:

‘[Children] used to eat lunch at home every afternoon but now that is no longer the case… [this] helps parents… feed their families because the afternoon meals are shifted to evening. We don’t consume as much food like we used to do in the past’ (Caregiver from Upper East Region); and

‘[Food preparation at home] has reduced. I used to cook for instance five pieces of yam, but now I cook just three, since the child is not hungry. There is no need to cook more’ (Caregiver from Ashanti Region); and

‘The truth is that because we don’t have money, the small [amount] that we have is what we cook at home for all our school children… if they come and are not satisfied at school, we eat together but if they are satisfied, we enjoy our home meal alone’ (Caregiver from Northern Region).

More than 80 percent of recipients of free school meals from the survey sample, however, continued to eat the same amount of food at home. A range of reasons were provided in the focus group discussions to explain this result. Some caregivers cited parental responsibility to feed their children, while others expressed concern that school meals might be not sufficient to “carry” them until the evening. Some also wanted to ensure that their children were provided with a balanced diet, especially if children found the food given them at school to be “boring”. For example,

*‘This feeding programme is to encourage the children to come to school so that does not mean that it should become the opportunity for parents to neglect the children so for that matter we still have to prepare food for them because that food will not be sufficient for them to take them throughout the whole day’ (Caregiver from Northern Region);* and

‘We also serve them our food to vary the food they eat because sometimes they can come and eat rice in the school here so when they get back home we prepare [meals] to vary the food and make it balanced’ (Caregiver from Northern Region).

Other predictors of having breakfast and lunch at home were identified in the quantitative analysis. The convenience dimension of the school food environment as reflected by commuting distance to and from school was a significant and positive predictor of having meals at home as hypothesized (p<0.001) and as shown in TABLE 4. The importance of this factor in dietary behaviours was also highlighted by a caregiver in the focus group discussions:

‘Some of the children, on their way home, they get scorched by the burning sun to the extent that when they reach home, they may be extremely tired and hungry, and some may even sleep and wake up later to eat’ (Caregiver from Duna, Northern Region).

TABLE 5 shows that adolescents were also less likely to have meals at home (coeff=-0.08, p<0.01). Furthermore, compared to southern Ghana, children and adolescents from northern Ghana were generally more likely to have breakfast before school or lunch at home, which may reflect more traditional values. Household size was negatively associated with the frequency in going home for lunch.

#### 3.2.4 Dietary behaviours – taking food to school

TABLE 6 presents the multi-level regression results for taking food to school. Older adolescents were less likely (coeff=-0.03, p<0.05) to take food to school while children and adolescents in urban areas were substantially more likely to bring food (coeff=0.49, p<0.001).

#### 3.2.5 Dietary behaviours - spending money on food at or near school.

TABLE 6 also presents the findings from the regression model investigating factors associated with the amount of money brought to school. This analysis was restricted to the sub-sample of children and adolescents who reported bringing money to school at least once in the past week. The results indicate that free school meal receipt was negatively associated with bringing money to school (coeff=-0.07, p<0.001) as well as the amount of money brought to school (coeff=-0.08, p<0.01) In the focus group discussions, caregivers reported giving amounts of money ranging between 20 pesewas and one cedi to their children every school day. In line with the findings from the regression analysis, some caregivers reported giving less money to their children on account of the provision of free school meals, thereby making financial savings. For example,

*‘I have reduced the money I give to my children in the morning from one Ghana cedis to fifty Ghana pesewas’ (Caregiver from Volta region);* and

‘Yes, it has helped financially. Previously we had to give them money to buy something when it is getting to midday but because the feeding is now free, we are able to save some money. We would be liars if we said it hasn’t benefited us in any way’ (Caregiver from Central Region).

Others reported that less obligation was felt to give money to children if household resources were limited as illustrated by the quote below:

‘Before the school feeding programme, you had to go and borrow money if you didn’t have for the child to buy something, but now the programme has reduced the burden such that even when there is no money, the child can fall back on what will be served in school’ (Caregiver from Volta region).

Other caregivers reported that they still gave the same amount of pocket money after the provision of the free school meals while acknowledging that the children’s needs were better ensured. For example,

‘Before the school feeding programme, I could give them money and it still wouldn’t be enough. But now, everything seems to be going smoothly’ (Caregiver from Central Region); and

‘I still give each [child] one cedi. They tell me they buy pencil and other things in school, reducing the amount will not be enough for them to buy all the daily items they need. (Caregiver from Volta Region).

The discussions with the caregivers suggested that supplementing breakfast could be one reason for the continued provision of money to children and adolescents. For example,

*‘In the morning, my children also normally take porridge. I also give them money when they are going to school so that they can buy something else to eat when they are hungry’ (Caregiver from Central Region);* and

‘In the morning… after they take porridge, you get them either 10 or 20 pesewas for them to buy something in the school so that they can learn properly, and when they come home, we then cook for them to eat. But now… with [school feeding], we have stopped giving them money to go to school’ (Caregiver from Northern Region).

The availability of foods for sale, in particular meals (p<0.001) and sugar-sweetened beverages (p<0.05), was also positively associated with bringing money to school in the regression analysis (see TABLE 5). Caregivers of children who received free school meals gave some indication in the focus group discussions that their children bought snacks and sweets rather than proper meals. For example,

‘I give 50 pesewas to each one of my children. But it impossible to tell exactly what they buy… sometimes when try to find out how they spent the money, they give you conflicting responses. As one says he/she bought water and kanfer^4^, the other exposes that they buy ice cream or toffee’ (Caregiver from Volta Region).

The survey analysis uncovered differences in expenditures by age. For example, older adolescents (aged 15 to 17 years) spent on average GHS 0.65 ($0.33) more than children (aged 5-10 years) in the past week (p<0.001). Children and adolescents from urban areas were more likely than rural children and adolescents to bring food or money to school, and the amount of money spent was greater (p<0.05). The multi-level regression results also indicate that household asset score was predictive of bringing money to school, as was being in the southern and wealthier districts of the country.

Child gender and having a younger sibling less than five years of age did not emerge as significant in any of the regression models, nor did interactions between child gender and age. In the focus group discussions, however, some caregivers noted that older or bigger children received more food, and a pro-boy bias when distributing food within the household. Alternative thresholds for the age groups of children, young adolescents and older adolescents did not alter the statistical significance and sign of the results presented in TABLES 5 and 6. The use of distance to school reduced the sample size considerably, but did not change the sign and statistical significance of findings except for money brought to school. Binary constructions of the dietary behavior measures did not affect the statistical significance of findings.

## 4. DISCUSSION

The study contributes to a presently small evidence base on school food environments and dietary behaviours in low- and middle-income countries. Ghana provides an interesting case as it was a low-income country at the time of the study undergoing the nutrition transition. The school food environment is largely defined by the school feeding programme, which seeks to procure nutritious, locally grown foods. The programme’s sustainability is protected by a School Feeding Policy, which was launched in 2016. This policy can provide a platform for enhancing the school food environment resulting in the promotion of healthy food choices among children and adolescents (de Villiers and Faber 2016).

Some results are consistent with previous evidence. For example, findings related to skipping breakfast or buying unhealthy foods from vendors echo the South African studies mentioned earlier (Faber et al 2014; Claasen et al 2016; van der Berg and Meko 2016). In addition, this study found that more than half of the sampled schools in Ghana offered foods for sale such as sugar-sweetened beverages by independent vendors. Private vendors may be crowded out due to a reduced competitive edge in the presence of the GSFP or a lower demand or desirability demonstrated by children and adolescents, following, for instance, nutrition education programmes. The availability of these foods may offset the benefits of free school meals for children and adolescents attending schools that participate in the GSFP, especially if they continue to bring pocket money to school to buy food. The amount of money brought to school was low in comparison with findings from another study reporting that mean daily expenditure of adolescents on ready to eat foods was GHS 2.18 ($1.09) in the Brong Ahafo Region, and GHS 1.43 ($0.72) in the Northern Region, translating to an estimated GHS 10.90 and GHS 7.15 per week respectively (Armar-Klemesu, M et al, 2014). Out-of-home consumption is an established risk factor for higher energy and fat intake, and low micronutrient intake (Lachat et al. 2012).

Regularity in the provision of school meals is also critical for establishing healthy and consistent dietary behaviours (Bundy et al. 2009). In Ghana, the current per-meal subsidy of 50 pesewas ($0.25) is low relative to the cost of food, which may lead to irregular provision, or meals of lower quality and quantity, especially if the currency value fluctuates significantly (Parish and Gelli 2015). Children and adolescents may respond to irregular meal provision by going home for lunch or spending money on food at or near school.

Household income is also important, reflecting the affordability dimension of the school food environment. The provision of free meals at school may be especially important for households from lower socioeconomic strata in rural areas. The targeting of the GSFP to the poorest and most vulnerable households may strengthen the role of the school food environment for those in greatest need, and introducing cost recovery mechanisms can support the financial sustainability of the programme (Alderman and Bundy 2012).

Commuting time to and from school was significantly associated with the frequency in going home for lunch and spending money on food at or near school. Other factors related to convenience such as product placement and food environments around schools, particularly in urban settings, could affect the diet quality of youth as has been investigated in some high-income countries (Hirschman and Chiriqui 2013; Adamson et al. 2013). Thus, the school food environment may contribute not only to school-based behaviours, but also in its vicinity, as argued by Van Der Berg and Meko (2016).

School-based nutrition education or behavioural change communication could promote internal and external factors related to the desirability of school meals and promote lifelong healthy eating (Silveira et al. 2011). This is especially important for adolescents, who have more autonomy regarding food purchase and consumption decisions and who may be differentially targeted or impacted by factors such as social norms and advertising (Mallick et al. 2014; Cusatis and Shannon 1996). This study indicates that adolescents were less likely to have breakfast or lunch at home, while they were more likely to bring money to school to buy food. Household SES and age may influence the desirability of foods for sale, resulting in more money spent on food at or near school.

### 4.1 Limitations

There were several limitations to the study. The focus was on the availability dimension of food environments due to the information available from the data, and were based on perceptions not observation. The other three dimensions – affordability, convenience and desirability – which may be more easily explored through qualitative studies can hold significant influence on child and adolescent dietary behaviours as well (Verstraeten et al. 2014). Furthermore, information on child and adolescent dietary behaviours was not collected directly at the child or adolescent level, but reported by the head of household.

As the household questionnaire was conducted at the homes of respondents in the community, some of the children and adolescents in these households may not have been pupils at the schools sampled in the school questionnaire. An analysis of the 2011-2012 EMIS data noted an average of 1.4 schools per community suggesting that in most cases the sampled children and adolescents did in fact attend the sampled school. In comparing responses from the household and school surveys, a high correlation (r=0.77) was noted between school participation in GSFP as reported in the school survey and household reporting of free school receipt in the household survey. Differences in reporting may also be due to the regularity in the provision of meals through the GSFP. Schools may indeed be part of the GSFP but not providing meals due to delays in payments to caterers, which was a significant concern at the time of the data collection.^5^

While the survey data was not nationally representative, the sample drew from all 10 regions of Ghana. Due to the targeting of the GSFP to more food insecure areas, in particular the north and rural areas of Ghana, it is likely that children and adolescents who attended schools that offered GSFP were from households of lower SES than children and adolescents who attended schools without GSFP. The focus group discussions concentrated on the northern regions of Ghana, and may not fully reflect the range of household responses to the school feeding programme and dietary behaviours exhibited by the child or adolescent.

Furthermore, the cross-sectional nature of the survey data limits causal interpretations. The availability of foods for sale at school, as well as the types of foods available, may reflect market demand from children and adolescents who attend the school or vice versa. Analysis of both the baseline and endline of the impact evaluation in Ghana, as well as other studies that employ rigorous methods can provide more conclusive evidence.

## 5. CONCLUSIONS

The school food environment can provide policy avenues to promote nutritional outcomes for children and adolescents. Refining the concept of food environments and measurement approaches for different target populations such as children and adolescents is an important area for future work to inform effective policy design. The developing evidence base on nutrition value chains, which relate to both supply and demand pathways, can be useful for this purpose (Gelli et al, 2015). In addition, a food environments framework could be leveraged to other critical issues in school nutrition in low- and middle-income countries such as food safety. More investigation is also needed on how nutrition education and behavioural change communication delivered through schools can enhance diet quality. Promoting a healthy school food environment may be more effective in communities where norms already support healthy diets and before nutrition transitions have taken place (Herforth and Ahmed, 2015).

## Footnotes

1 Groundnut is the Ghanaian word for peanuts. Similarly, okro is other countries may be known as okra or lady’s fingers.

2 Data from the Ghana Education Service is available from the website: http://www.ghanaeducationdata.com/ (link downloaded November 28, 2015).

3 School food environment measures were effectively at the community level as there was one school per community in the data.

4 Kanfer is similar to abolo, which is ground, soaked corn flour with sugar that is steamed or baked on leaves.

## ANNEX

**Table A.1:** Questions about the school food environment and dietary behaviours from the household and school questionnaires

Household Questionnaire	
Question	Response Options
Does [NAME] receive free meals at school?	1= Yes 2= No
*If response = ‘yes’, then continue with the following questions:*	
For how many of the past 7 days has [NAME] received a free meal or snack at school?	0-5 (Only include days from Monday to Friday)
Does [NAME] eat less food at home on days (he / she) eats a free meal or snack at school?	1= Yes 2= No
Does [NAME] ever bring food home from the free meal for other household members?	1= Yes 2= No
For how many of the past 7 days did [NAME] eat breakfast before school?	0-5 (Only include days from Monday to Friday)
For how many of the past 7 days did [NAME] take food to school for lunch and / or snacks?	0-5 (Only include days from Monday to Friday)
For how many of the past 7 days did [NAME] come home to eat for lunch?	0-5 (Only include days from Monday to Friday)
For how many of the past 7 days did [NAME] spend money on food at or near school? Include any money given to the school administration to buy food for [NAME] excluding school feeding.	0-5 (Only include days from Monday to Friday)
How much money did [NAME] spend on food at or near school in the last 7 days?	Ghc (only include days from Monday to Friday)

School Questionnaire	
Question	Response Options
What types of foods are currently sold in your school?	[multiple responses are applicable] Prepared meals Confectionery (Biscuits, pastries, etc) Sugar sweetened beverages Fruits Other (please specify)
Is this school currently part of the Ghana School Feeding Programme?	Yes/No
